# Emergency Department and Inpatient Healthcare utilization due to Hypertension

**DOI:** 10.1186/s12913-016-1563-7

**Published:** 2016-07-26

**Authors:** Jasvinder A. Singh, Shaohua Yu

**Affiliations:** 1 Medicine Service, Birmingham VA Medical Center, Birmingham, AL USA; 2 Department of Medicine at School of Medicine, and Division of Epidemiology at School of Public Health, University of Alabama at Birmingham, Birmingham, AL USA; 3 Department of Orthopedic Surgery, Mayo Clinic College of Medicine, Rochester, MN USA

**Keywords:** Emergency department, Hospitalization, Health care utilization, Charges, Hypertension, Predictors, Hospital discharge, Predictors

## Abstract

**Background:**

Hypertension is one of the commonest chronic diseases, yet limited data are available for related health care utilization. Our study objective was to describe the emergency department (ED) and subsequent hospitalization related health care utilization and charges due to hypertension in the U.S.

**Methods:**

We used the National ED sample (NEDS) to study hypertension-related utilization and charges. Multivariable-adjusted linear or logistic regression was used to assess hypertension-associated ED and hospitalization outcomes (disposition, length of stay, charges), adjusted for patient demographic, comorbidity and hospital characteristics.

**Results:**

There were 0.92, 0.97 and 1.04 million ED visits (0.71–0.77 % of all ED visits) with hypertension as the primary diagnosis in 2009, 2010 and 2012, respectively; 23 % resulted in hospitalization. ED charges were $2.00, $2.27 and $2.86 billion, and for those hospitalized, total charges (ED plus inpatient) were $6.62, $7.09 and $7.94 billion, in 2009, 2010 and 2012, respectively. Older age (50 to 65 years), female sex, metropolitan area residence, South or West U.S. hospital location, private insurance and the presence of congestive heart failure were each associated with higher charges for an ED visit with hypertension as the primary diagnosis. Younger age, metropolitan residence, Medicaid insurance, hospital location in the Northeast and co-existing diabetes, gout, coronary heart disease, chronic obstructive pulmonary disease, hyperlipidemia and osteoarthritis were associated with higher risk, whereas male sex was associated with lower risk of hospitalization after ED visit for hypertension. In 2012, 71.6 % of all patients hospitalized with hypertension as the primary diagnosis were discharged home. Older age, metropolitan residence and most comorbidities were associated with lower odds, whereas male sex, payer other than Medicare, South or West U.S. hospital location were associated with higher odds of discharge to home.

**Conclusions:**

Hypertension is associated with significant healthcare burden in the U.S. Future studies should assess strategies to reduce hypertension-associated cost and health care burden.

**Electronic supplementary material:**

The online version of this article (doi:10.1186/s12913-016-1563-7) contains supplementary material, which is available to authorized users.

## Background

Hypertension is one of the commonest chronic diseases. It affects 32.5 % of U.S. adults [1]. Hypertension is the primary diagnosis for 38.9 million physician office visits annually in the U.S. [1]. Hypertension and related renal disease are responsible for 27,853 deaths annually [1]. Hypertension cost the U.S. approximately $49.9 billion in 2010, $29.5 billion in direct health care expenditures and $20.4 billion in indirect costs [1, 2]. Thus, as a chronic condition, hypertension is associated with high public health and cost burden.

Hypertension is usually accompanied by other diseases such as heart disease, renal failure, stroke and vascular disease [3], conditions that have significant associated morbidity and mortality [4]. Suboptimal treatment of hypertension may not only increase the risk of accompanying diseases, but also lead to higher health resource utilization. With an increasing focus and attention on improving the health of populations and reducing unnecessary health care utilization, current knowledge of resource utilization associated with hypertension is needed.

To our knowledge, little is known about ED and inpatient utilization related to hypertension in the U.S. A recent study drew attention to time-trends in ED visits with hypertension as the primary diagnosis, and found a 4 % increase per year from 2006 to 2012 [5]. This study focused on ED visits by age and co-existing comorbidities and examining all hypertension-related visits, with or without hypertension as the primary diagnosis. No analyses were performed assessing charges or the predictors of ED or inpatient resource utilization after ED visits with hypertension as the primary diagnosis [5]. Thus, knowledge gaps exist in this area, that need to be addressed. We recently performed analyses for utilization related to COPD and gout-related ED visits in the U.S. and important predictors, including ED disposition, ED and total hospital charges and predictors of ED disposition and hospitalization outcomes [6, 7]. Using the same approach, we investigated healthcare utilization, charges and outcomes in ED visits due to hypertension. In this study, our aims were to: (1) describe the hypertension-related ED and inpatient utilization and related charges in the U.S.; and (2) assess whether patient, comorbidity or hospital characteristics were predictors of ED- and inpatient resource utilization due to hypertension.

## Methods

### Data source and study population

This study was performed using the discharge data from the Nationwide Emergency Department Sample (NEDS) [8, 9]. NEDS is the largest, publically available, all-payer U.S. ED database that contains a 20-percent stratified sample of ED visits from across the U.S. The data are provided by the Agency for Healthcare Research and Quality’s Healthcare Cost and Utilization Project (HCUP) State Emergency Department Databases (SEDD) and the State Inpatient Databases (SID) [8, 9] that capture discharge information on ED visits that do not result vs. that result in hospitalization, respectively. Thirty states, including 950 U.S. hospitals, contributed data regarding 31 million ED visits in 2012, which were weighted to calculate the national estimates related to 134 million ED visits in the U.S. [9].

We identified hypertension related visits in people aged 18 and over using the International Classification of Diseases, Common Modification (ICD-9-CM) code of 401.xx, 402.xx, 403.xx, 404.xx, and 405.xx. This approach has been shown to be valid with a positive predictive value >95 % [10, 11]. Visits are categorized as those with 1) hypertension as the primary diagnosis (where hypertension was listed as the first/primary diagnosis), and (2) hypertension as primary or secondary diagnosis. We used the data from 2009, 2010 and 2012, since 2011 data were not available for analyses, due to data duplication issues.

### Outcomes of interest and covariates

In this study, we examined several outcomes of interest. These included outcomes related to ED discharge disposition (hospitalization, discharge to home etc.) and ED charges. We also assessed outcomes in patients hospitalized with hypertension as the primary diagnosis, including the factors associated with discharge after hospital admission (to home vs. other; to nursing home/skilled nursing facility vs. other), hospital stay (total duration; hospital stay >2 days vs. ≤ 2 days) and total charges (inpatient + ED).

NEDS includes reasons for ED visit (diagnoses and procedures); up to 15 ICD-9-CM codes are listed in primary (first) or secondary positions (2–15). We specified several common comorbidities in secondary position as covariates of interest, including hyperlipidemia, coronary heart disease (CHD), renal failure, heart failure (HF), chronic obstructive pulmonary disease (COPD), diabetes, gout and osteoarthritis. Patient characteristics including age, sex, insurance status, residence [urban vs. rural], annual median household income estimated using residential zip code were assessed. We also examined hospital characteristics including geographical region, location in metropolitan vs. non-metropolitan area, and teaching vs. non-teaching status, as covariates.

### Statistical analysis

With the exception of descriptive analyses (overall charges, number of ED visits, hospital stay), which we calculated for ED visits due to hypertension (hypertension as the primary diagnosis) as well as for hypertension-related ED visits (hypertension as the primary or secondary diagnosis), all other analyses were limited to visits with hypertension as the primary diagnosis. Appropriate weights provided by NEDS were used to obtain weighted national estimates for 2009, 2010 and 2012.

We used the 2012 NEDS data to analyze whether pre-specified patient and hospital factors were associated with outcomes of interest in patients with hypertension as the primary diagnosis for ED visits and in patients with inpatient admission with hypertension as the primary diagnosis. We performed multivariable-adjusted logistic regression (discharge disposition from ED and from the hospital) or linear regression (ED charges, length of hospital stay, total charges [inpatient + ED]) using SAS version 9.3 (SAS corporation, Cary, NC, USA). Analyses were adjusted for important confounders and covariates including patient characteristics, comorbidities and hospital characteristics, as listed in the section above. These analyses used the actual NEDS sample, without extrapolation to national estimates.

Sensitivity analyses were performed with log of hospital duration of stay and total charges (ED plus inpatient), since log variables were more normally distributed than the original variables. We also examined factors associated with short vs. longer hospital (≥2 days) to better understand inpatient utilization due to hypertension.

## Results

### Clinical and demographic characteristics

Hypertension was associated with high health care utilization in the ED in the U.S., which also seemed to have increased over the study period. The number of ED visits for hypertension as the primary diagnosis were 0.92 million in 2009, 0.97 in 2010 and 1.04 million in 2012 (Table 1), a 13 % increase in 4 years. The mean age for patients with ED visit with hypertension in 2012 was 59 years, 57 % were female and 15 % were in the highest income quartile (≥$63,000) (Table 1). In 2012, Medicare was the primary payer for 43 %, and almost half of all ED visits for hypertension occurred in hospitals located in the Southern U.S, with only 16 % each in Northeastern and Western U.S and 20 % in the Midwest. Patient characteristics were similar across study years including sex, residence, the primary payer, hospital region and teaching status etc. (Table 1).

**Table 1:** Emergency department (ED) visits for hypertension as the primary diagnosis in year 2009, 2010 and 2012 NEDS database^a^

	2009 NEDS	2010 NEDS	2012 NEDS
ED visits	920,984 (0.71)	972,631 (0.75)	1,041,223 (0.77)
Age, in years			
Mean (SE)	58.64 (0.23)	58.43 (0.22)	59.17 (0.22)
Median (IQR)	56.96 (45.44, 71.73)	56.79 (45.35, 71.28)	57.95 (46.29, 71.98)
Sex			
Female	530,143 (57.62)	558,253 (57.40)	595,212 (57.17)
Patient location (residence)			
Micropolitan/not metro	169,247 (18.51)	175,858 (18.19)	184,852 (17.84)
Metropolitan (large or small)	744,925 (81.49)	791,005 (81.81)	851,485 (82.16)
Median house hold income			
1st quartile (< $38,999)	340,766 (37.96)	368,468 (38.80)	389,354 (38.19)
2nd quartile ($39,000 to $47,999)	250,997 (27.96)	252,070 (26.55)	255,324 (25.04)
3rd quartile ($48,000 to $62999)	179,959 (20.05)	186,460 (19.64)	219,568 (21.53)
4th quartile ($63,000 or more)	125,880(14.02)	142,564 (15.01)	155,369 (15.24)
Primary payer			
Medicare	370,933 (40.37)	391,925 (40.41)	443,993 (42.69)
Medicaid	106,282 (11.57)	119,606 (12.33)	138,606 (13.33)
Private insurance	244,669 (26.63)	247,195 (25.49)	229,988 (22.12)
Self-pay/No charge	167,334 (18.32)	182,153 (18.78)	189,031 (18.18)
Other	28,560 (3.11)	29,001 (2.99)	38,337 (3.69)
Hospital Region			
Northeast	148,302 (16.10)	168,618(17.34)	168,303 (16.16)
Midwest	183,785 (19.96)	207,731 (21.36)	219,784 (21.11)
South	448,654 (48.71)	447,419 (46.00)	490,154 (47.07)
West	140,244 (15.23)	148,863 (15.31)	162,982 (15.65)
Teaching status of hospital			
Metropolitan non -teaching or non-metro	555,998 (60.37)	557,804 (57.35)	576,146 (55.33)
Metropolitan teaching	364,986 (39.63)	414,827 (42.65)	465,077 (44.67)

^a^Data shown are n (%), unless specified otherwise

*SE* standard error, *IQR* inter-quartile range

### ED and inpatient charges for Hypertension-visits (primary) and hypertension-related (primary or secondary) visits

ED visits with hypertension as the primary diagnosis constituted 0.71–0.77 % of all ED visits. The proportions of all ED visits that were due to hypertension as the primary diagnosis were stable across the study years of 2009, 2010 and 2012 at 0.71, 0.75 and 0.77 %; however, the respective ED charges increased and were $2.00, $2.27 and $2.86 billion, respectively (Table 2). We also noted a similar increase in the total charges for ED and inpatient services in patients who were hospitalized with hypertension as their primary diagnosis at $6.62, $7.09 and $7.94 billion, respectively (Table 2).

**Table 2:** Descriptives for the main ED visit-related outcomes with hypertension diagnosis

	2009	2010	2012
Hypertension visits (hypertension as the primary diagnosis)			
All Hypertension ED visits with hypertension as primary diagnosis weighted n (% of total ED visits))	920,984 (0.71)	972,631 (0.75)	1,041,223 (0.77)
Total ED charges ($)	1,997,899,801	2,269,712,249	2,859,937,626
All Hypertension ED visits (not admitted to the same hospital) with hypertension as primary diagnosis	681,136	725,960	798,826
Total ED charges ($)	1,618,467,684	1,836,308,560	2,359,979,640
All Hypertension inpatient admissions with hypertension as primary diagnosis for those with ED visits	239,847	243,071	242,397
Total inpatient charges ($)	5,770,718,820	6,295,295,829	7,051,813,524
Duration of hospital stay, in days	4.13 (0.05)	4.02 (0.05)	3.98 (0.05)
Total charges for ED and inpatient services ($)	6,624,334,293	7,092,082,567	7,941,168,117
Hypertension-related visits (hypertension as the primary or secondary diagnosis)			
All Hypertension inpatient admissions with hypertension in any position for those with ED visits	10,257,840	10,504,104	10,333,391
Total inpatient charges ($)	307,283,855,040	328,431,819,768	360,997,014,585
Duration of hospital stay, in days mean (SE)	4.93 (0.04)	4.81 (0.03)	4.72 (0.04)
Total charges for ED and inpatient services ($)	340,703,897,760	364,460,896,488	406,133,266,473

*SE* standard error

For the three study years, 2009, 2010 and 2012, of the ED-visits for hypertension as the primary diagnosis, 26, 25 and 23 % were hospitalized, respectively (Additional file 1). Respective mean ED charges for visits with hypertension as the primary diagnosis were $2,169, $2,334 and $2,747 per ED visit. Respective mean total charges (ED plus inpatient) in those hospitalized with hypertension as the primary diagnosis were $27,619, $29,177 and $32,761. The mean length of hospital stay was 4.1, 4.0 and 4.0 days, respectively (Additional file 1).

Total ED and inpatient charges with hypertension diagnosis in any position (primary or secondary), i.e. hypertension-related visits, were $406 billion in 2012, higher than the $341 billion in 2009 (Table 2).

### ED charges and discharge disposition for visits due to hypertension as the primary diagnosis

We used multivariable-adjusted linear regression adjusting for patient demographic, comorbidities and hospital characteristics to assess factors associated with higher charges for an ED visit with hypertension as the primary diagnosis. We found that age <50 years, female sex, residence in metropolitan area, private insurance, presence of HF and hospital location in South or West U.S. location were each associated with higher charges for an ED visit with hypertension as the primary diagnosis (Additional file 2).

Multivariable-adjusted logistic regression showed that younger age, metropolitan residence, Medicaid insurance and hospital location in the Northeast were associated with higher risk of hospitalization after ED visit for hypertension as the primary diagnosis (Table 3); male sex was associated with lower risk of hospitalization for hypertension. Patients with co-existing diabetes, gout, CHD, COPD, hyperlipidemia, renal failure, HF and osteoarthritis had a significantly higher adjusted odds of being admitted compared to patients without each of these conditions (Table 3). Unadjusted estimates for patients with ED-visit with hypertension as the primary diagnosis who were and were not subsequently admitted to the hospital are shown in Additional file 3.

**Table 3:** Predictors of hospital admission among patients presenting to ED with hypertension as the primary diagnosis

	Univariate	Multivariable-adjusted
	Odds ratio^a^ (95 % CI)	Odds ratio^a^ (95 % CI)
Age		
<50	Ref	Ref
50- <65	1.33 (1.28, 1.38)***	0.90 (0.86, 0.94)***
65- <80	1.60 (1.52, 1.68)***	0.74 (0.69, 0.79)***
≥80	2.26 (2.12, 2.40)***	0.91 (0.83, 0.98)*
Gender		
Female	Ref	Ref
Male	1.19 (1.15, 1.23)***	0.93 (0.89, 0.97)**
Median household income		
1st quartile (< $38,999)	Ref	Ref
2nd quartile ($39,000 to $47,999)	0.95 (0.88, 1.02)	0.96 (0.88, 1.04)
3rd quartile ($48,000 to $62999)	1.00 (0.91, 1.10)	0.95 (0.85, 1.06)
4th quartile ($63,000 or more)	1.11 (1.00, 1.25)	0.95 (0.84, 1.09)
Primary payer		
Medicare (ref)	Ref	Ref
Medicaid	0.79 (0.74, 0.85)***	1.14 (1.02, 1.27)
Private insurance	0.45 (0.43, 0.47)***	0.99 (0.92, 1.07)
Self-pay/No charge	0.36 (0.33, 0.40)***	0.96 (0.87, 1.06)
Other	0.56 (0.47, 0.66)***	1.21 (1.01, 1.44)*
Patient location (residence)		
Micropolitan/not metro	Ref	Ref
Metro (large or small)	1.84 (1.65, 2.04)***	1.68 (1.50, 1.90)***
Hospital Region		
Northeast	Ref	Ref
Midwest	0.75 (0.62, 0.91)*	0.62 (0.51, 0.75)***
South	0.75 (0.63, 0.88)*	0.67 (0.54, 0.81)***
West	0.73 (0.61, 0.88)***	0.51 (0.40, 0.65)***
Teaching status of hospital		
Metropolitan non-teaching or non-metro	Ref	Ref
Metropolitan teaching	1.37 (1.22, 1.54)***	1.04 (0.89, 1.21)
Comorbidities		
CHD (ref: no)	7.27 (6.84, 7.72)***	2.80 (2.64, 2.97)***
Hyperlipidemia (ref: no)	4.90 (4.55, 5.26)***	3.27 (3.03, 3.54)***
Renal failure (ref: no)	14.08 (12.83, 15.46)***	7.52 (6.86, 8.25)***
Heart Failure (ref: no)	22.38 (20.58, 24.34)***	8.55 (7.86, 9.30)***
Gout (ref: no)	5.52 (4.96, 6.15)***	1.86 (1.60, 2.18)***
Diabetes (ref: no)	3.57 (3.42, 3.73)***	1.51 (1.44, 1.58)***
COPD (ref: no)	5.62 (5.24, 6.02)***	2.09 (1.94, 2.24)***
Osteoarthritis (ref: no)	5.64 (4.92, 6.45)	3.76 (3.18, 4.44)***

CHD, coronary heart disease; COPD, chronic obstructive pulmonary disease; OR, odds ratio; CI, confidence interval

^a^Odds ratio from the logistic regression

*p-value <0.05; **p-value <0.01; ***p-value <0.001; Significant odds ratios are in bold

### Hospitalization disposition and predictors for Hypertension-visits (primary)

In 2009, 2010 and 2012, 73.6 %, 73.1 % and 71.6 % of all patients hospitalized with hypertension as the primary diagnosis were discharged home (Additional file 4), respectively. Discharge disposition to other settings after hospitalization for hypertension did not vary by the study year.

Multivariable-adjusted analyses showed that older age, metropolitan residence, CHD, renal failure, HF, diabetes, COPD and osteoarthritis were associated with lower odds, whereas male sex, payer other than Medicare, South or West U.S. hospital location, hyperlipidemia and gout were associated with higher odds of discharge to home (Table 4).

**Table 4:** Predictors of Discharge to home among patients who had a hospital admission after presenting to ED with hypertension as the primary diagnosis

	Univariate	Multivariable-adjusted
	Odds Ratio^a^ (95 % CI)	Odds Ratio^a^ (95 % CI)
Age		
<50	Ref	
50- <65	0.62 (0.58, 0.68)***	0.74 (0.68, 0.81)***
65- <80	0.28 (0.25, 0.30)***	0.48 (0.43, 0.53)***
≥80	0.12 (0.11, 0.13)***	0.21 (0.18, 0.23)***
Gender		
Female	Ref	Ref
Male	1.28 (1.22, 1.34)***	1.06 (1.00, 1.11)*
Median house hold income		
1st quartile (< $38,999)	Ref	Ref
2nd quartile ($39,000 to $47,999)	0.96 (0.89, 1.04)	1.05 (0.97, 1.14)
3rd quartile ($48,000 to $62999)	0.89 (0.80, 0.98)*	1.05 (0.95, 1.16)
4th quartile ($63,000 or more)	0.70 (0.64, 0.77)***	1.00 (0.90, 1.10)
Primary payer		
Medicare	Ref	Ref
Medicaid	2.67 (2.46, 2.90)***	1.39 (1.25, 1.55)***
Private insurance	4.29 (3.93, 4.68)***	2.07 (1.88, 2.27)***
Self-pay/No charge	7.23 (6.53, 8.01)***	2.71 (2.35, 3.12)***
Other	4.89 (3.92, 6.10)***	2.20 (1.76, 2.74)***
Patient location (residence)		
Micropolitan/not metro	Ref	Ref
Metropolitan (large or small)	0.97 (0.87, 1.09)	0.89 (0.80, 0.99)*
Hospital Region		
Northeast	Ref	Ref
Midwest	1.27 (1.08, 1.50)**	1.10 (0.94, 1.29)
South	1.56 (1.40, 1.74)***	1.45 (1.28, 1.64)***
West	1.64 (1.41, 1.91)***	1.80 (1.55, 2.09)***
Teaching status of hospital		
Metropolitan non -teaching or non-metro	Ref	Ref
Metropolitan teaching	1.15 (1.02, 1.29)*	1.15 (1.04, 1.28)
Comorbidities		
CHD (ref: no)	0.59 (0.56, 0.63)***	0.98 (0.93, 1.03)
Hyperlipidemia (ref: no)	1.05 (1.00, 1.10)*	1.28 (1.22, 1.35)***
Renal failure (ref: no)	0.52 (0.48, 0.55)***	0.75 (0.71, 0.80)***
Heart Failure (ref: no)	0.36 (0.34, 0.39)***	0.62 (0.59, 0.66)***
Gout (ref: no)	0.79 (0.73, 0.87)***	1.14 (1.03, 1.26)**
Diabetes (ref: no)	0.75 (0.71, 0.78)***	0.82 (0.78, 0.86)***
COPD (ref: no)	0.50 (0.47, 0.54)***	0.81 (0.75, 0.87)***
Osteoarthritis (ref: no)	0.62 (0.58, 0.67)***	0.94 (0.86, 1.02)
Length of stay, in days	0.84 (0.83, 0.86)***	0.88 (0.87, 0.89)***

CHD, coronary heart disease; COPD, chronic obstructive pulmonary disease; OR, odds ratio; CI, confidence interval

^a^Odds ratio from the logistic regression

*p-value <0.05; **p-value <0.01; ***p-value <0.001; Significant odds ratios are in bold

### Length of Hospital Stay and total charges for Hypertension-visits (primary)

Multivariable-adjusted linear regression showed that older age categories (50- <65, 65- <80 and ≥80, compared to age <50 years), metropolitan patient residence, metropolitan teaching hospital, Medicaid as primary payer and several comorbidities including renal failure, HF, diabetes, and COPD were associated with longer hospital stay for hypertension (Table 5). On the other hand, hyperlipidemia, osteoarthritis, private insurance and hospital location other than Northeast were associated with shorter hospital stay for patients admitted to the hospital with hypertension as the primary diagnosis (Table 5).

**Table 5:** Predictors of duration of hospital stay among patients with hypertension who were admitted to the hospital after presenting to ED with hypertension as the primary diagnosis

	Univariate	Multivariable-adjusted
	Beta-estimate^a^ (95 % CI)	Beta-estimate^a^ (95 % CI)
Age		
<50	Ref	Ref
50- <65	0.49 (0.37, 0.61)***	0.47 (0.35,0.59)***
65- <80	1.00 (0.86, 1.15)***	0.83 (0.66,1.01)***
≥80	1.13 (0.97, 1.29)***	0.89 (0.72,1.05)***
Gender		
Female	Ref	Ref
Male	0.17 (0.08, 0.26)**	−0.03 (−0.12,0.06)
Median house hold income		
1st quartile (< $38,999)	Ref	Ref
2nd quartile ($39,000 to $47,999)	0.06 (−0.09, 0.20)	0.09 (−0.04, 0.22)
3rd quartile ($48,000 to $62999)	0.07 (−0.10, 0.25)	0.06 (−0.10, 0.23)
4th quartile ($63,000 or more)	0.27 (0.07, 0.48)**	0.09 (−0.08, 0.27)
Primary payer		
Medicare (ref)	Ref	Ref
Medicaid	−0.10 (−0.27, 0.08)	0.47 (0.28, 0.66)***
Private insurance	−1.05 (−1.17, −0.93)***	−0.14 (−0.27, 0.00)*
Self-pay/No charge	−1.32 (−1.50, −1.14)***	−0.19 (−0.40, 0.03)
Other	−0.92 (−1.28, −0.56)***	0.03 (−0.33, 0.39)
Patient location (residence)		
Micropolitan/not metro	Ref	Ref
Metro (large or small)	0.36 (0.15, 0.56)**	0.17 (0.02, 0.32)*
Hospital Region		
Northeast	Ref	Ref
Midwest	−0.85 (−1.17, −0.53)***	−0.88 (−1.16, −0.60)***
South	−0.58 (−0.87, −0.29)**	−0.56 (−0.81, −0.31)***
West	−0.58 (−0.89, −0.26)**	−0.85 (−1.13, −0.56)***
Teaching status of hospital		
Metropolitan non -teaching or non-metro	Ref	Ref
Metropolitan teaching	0.24 (0.05, 0.44)*	0.19 (0.02, 0.35)*
Comorbidities		
CHD (ref: no)	0.54 (0.44, 0.65)***	−0.05 (−0.14, 0.05)
Hyperlipidemia (ref: no)	−0.51 (−0.60, −0.41)***	−0.59 (−0.68, −0.50)***
Renal failure (ref: no)	2.10 (1.95, 2.25)***	1.63 (1.48, 1.78)***
Heart Failure (ref: no)	2.07 (1.94, 2.20)***	1.53 (1.41, 1.65)***
Gout (ref: no)	−0.48 (−0.66, −0.31)***	0.13 (−0.05, 0.30)
Diabetes (ref: no)	0.57 (0.47, 0.67)***	0.11 (0.02, 0.21)*
COPD (ref: no)	1.01 (0.87, 1.15)***	0.29 (0.16, 0.42)***
Osteoarthritis (ref: no)	−0.16 (−0.32, −0.01)*	−0.16 (−0.30, −0.02)*

CHD, coronary heart disease; COPD, chronic obstructive pulmonary disease

^a^beta-estimate from linear regression

*p-value <0.05; **p-value <0.01; ***p-value <0.001; Significant estimates are in bold

In multivariable-adjusted analyses, older age, median household income in the highest quartile, metropolitan location and most comorbidities including CHD, renal failure, gout, HF, and COPD were associated higher total (ED + inpatient) hospital charges for hypertension hospitalizations (Table 6). Uninsured primary payer, Midwest U.S. hospital location, hyperlipidemia and osteoarthritis were associated with lower hospital charges for hypertension hospitalizations (Table 6).

**Table 6:** Predictors of total hospital charges (inpatient + ED charges) among patients with hypertension hospitalized after presenting to ED with hypertension as the primary diagnosis

	Univariate		Multivariable-adjusted	
	B-estimate^a^ (95 % CI)	P-value	B-estimate^a^ (95 % CI)	P-value
Age				
<50	Ref		Ref	
50- <65	**4099.5 (2790.9, 5408.1)**	**<0.0001**	**3478.3 (2007.5, 4949.1)**	**<0.0001**
65- <80	**7602.0 (5920.2, 9283.9)**	**<0.0001**	**4916.7 (3322.1, 6511.3)**	**<0.0001**
≥80	5465.2 (3421.7, 7508.6)	<0.0001	1419.5 (−346.3, 3185.3)	0.1149
Gender				
Female (ref)	Ref		Ref	
Male	**2634.0 (1658.1, 3609.9)**	**<0.0001**	273.2 (−603.8, 1150.2)	0.5410
Median household income				
1st quartile (< $38,999)	Ref		Ref	
2nd quartile ($39,000 to $47,999)	**2807.9 (573.2, 5042.6)**	**0.0139**	1551.8 (−519.5, 3623.2)	0.1418
3rd quartile ($48,000 to $62999)	**4816.3 (1994.3, 7638.3)**	**0.0008**	2010.2 (−454.3, 4474.7)	0.1097
4th quartile ($63,000 or more)	**8879.6 (4721.0, 13038.2)**	**<0.0001**	**4122.0 (447.4, 7796.7)**	**0.0280**
Primary payer				
Medicare	Ref		Ref	
Medicaid	109.4 (−2624.6, 2843.44)	0.9374	1633.7 (−1032.0, 4299.4)	0.2293
Private insurance	**−5403.9 (−6842.8, −3965.1)**	**<0.0001**	−343.7 (−1752.1, 1064.8)	0.6320
Self-pay/No charge	**−9279.1 (−11307.1, −7251.05)**	**<0.0001**	**−2197.7 (−4074.3, −321.0)**	**0.0218**
Other	**−5871.1 (−9599.0, −2143.1)**	**0.0021**	**−3775.8 (−6842.5, −709.2)**	**0.0159**
Patient residence				
Micropolitan/not metro	Ref		Ref	
Metropolitan (large or small)	**10950.6 (8508.3, 13392.9)**	**<0.0001**	**8651.8 (6361.1, 10942.5)**	**<0.0001**
Hospital Region				
Northeast	Ref		Ref	
Midwest	**−9957.2 (−16070.1, −3844.23)**	**0.0014**	**−9936.6 (−15814.5, −4058.8)**	**0.0010**
South	−5883.6 (−11886.2, 119.0)	0.0547	−5384.8 (−11298.4, 528.80)	0.0742
West	**14494.2 (7118.5, 21869.9)**	**0.0001**	**11992.9 (5047.4, 18938.3)**	**0.0007**
Teaching status of hospital				
Metropolitan non –teaching or non-metro	Ref		Ref	
Metropolitan teaching	−1801.1 (−5304.5, 1702.3)	0.3131	−1094.6 (−4385.9, 2196.7)	0.5140
Comorbidities				
CHD (ref: no)	**5309.5 (3931.5, 6687.6)**	**<0.0001**	**1403.8 (186.8, 2620.8)**	**0.0238**
Hyperlipidemia (ref: no)	**−3780.9 (−4842.8, −2719.0)**	**<0.0001**	**−3947.7 (−4872.1, −3023.3)**	**<0.0001**
Renal failure (ref: no)	**15161.4 (13324.7, 16998.1)**	**<0.0001**	**11217.3 (9614.5, 12820.1)**	**<0.0001**
Heart Failure (ref: no)	**16095.3 (14106.3,18084.2)**	**<0.0001**	**11954.3 (10060.2, 13848.5)**	**<0.0001**
Gout (ref: no)	**−2037.1 (−3945.0, −129.2)**	**0.0364**	**2219.1 (520.7, 3917.6)**	**0.0105**
Diabetes (ref: no)	**3422.1 (2450.1, 4394.2)**	**<0.0001**	−661.5 (−1662.0, 338.9)	0.1946
COPD (ref: no)	**7870.4 (5955.5, 9785.3)**	**<0.0001**	**2955.0 (1137.8, 4772.2)**	**0.0015**
Osteoarthritis (ref: no)	**−4400.6 (−6279.1, −2522.1)**	**<0.0001**	**−2858.6 (−4473.5, −1243.7)**	**0.0005**

CHD, coronary heart disease; COPD, chronic obstructive pulmonary disease; Significant beta coefficients are in bold

^a^Beta-estimate from linear regression

We performed several sensitivity analyses to examine whether our findings were robust or not. Sensitivity analyses examining the log of hospital stay (Additional file 5), hospital stay dichotomized at 2 days (Additional file 6), or the log of total hospital charges (Additional file 7) showed similar results as the analyses above, and supported the robustness of our findings.

## Discussion

Our study provided contemporary national estimates of ED and inpatient utilization and charges for patients with hypertension as the primary diagnosis. We found that hypertension was the primary diagnosis (i.e., main reason) for over 1 million ED visits in 2012, which were roughly 0.7–0.8 % of all ED visits. Of these ED visits in 2012, 23 % resulted in hospitalization. We also examined the factors associated with health resource utilization and charges for ED and inpatient visits due to hypertension, i.e. with hypertension as the primary diagnosis.

We noted a 13 % increase in ED utilization due to hypertension in 4-years in contrast to decreasing utilization for hypertension reported recently in a Canadian study [12]. Differences in study findings may be due to differences in health care systems (multiple insurer system vs. single-payer Government system), country setting (U.S. vs. Canada) and the study time period (2009-2012 vs. 1997-2004). It is reassuring that our estimates of 13 % increase over 4 years (i.e. 3–4 % per year) and the overall number of ED visits almost replicate the 4 % annual increase reported in the previous 6-year NEDS study of ED visits with a primary diagnosis of hypertension, with slight difference due to inclusion of an additional code 437.2 in the earlier study [5].

Our study defines the public health burden and health care burden of hypertension in the emergency departments and hospitals in the U.S. To our knowledge, there is a lack of nationally representative studies assessing the burden of hypertension in the U.S., except a recent descriptive study [5]. These were ED visits with a primary diagnosis of hypertension and/or uncontrolled or untreated hypertension with other non-specific associated symptoms such as headache, dizziness, pedal edema, worsening of heart failure etc. These visits are unlikely to be due to stroke, myocardial infarction (MI) in the setting of hypertension urgencies or emergencies [13], since visits with these acute conditions would likely have stroke or MI as the primary diagnoses, not hypertension.

We found that of the ED-visits with hypertension as the primary diagnosis, 23 % resulted in hospitalizations. This admission rate was higher than that for gout-related ED visits at 7.7 % [7] and lower than the 49 % for COPD-related ED visits in the U.S. [6], analyzed for the same years using similar methodology and analyses, as this study. We planned these analyses *a priori* to be similar to our previous analyses of patients with COPD or gout to allow across-disease comparability of estimates and predictors of national U.S. healthcare utilization data. Of those hospitalized, 71.6 % were discharged home. The mean 2012 ED and inpatient charges were $2,747 and $32,761 per visit respectively and the mean length of hospital stay was 4.0 days. Total 2012 charges for ED and inpatient visits with hypertension as the primary diagnosis were $2.86 billion and $7.94 billion, respectively. These charges are comparable to total charges nationally for diabetic foot at $8.78 billion in 2010 [14], higher than changes for constipation at $1.62 billion [15], but lower than those for COPD at $14.2 billion in 2012 [6]. The mean ED visit charges of $2,747 for hypertension is slightly higher than that ED visits for diabetic foot at $2,324 [14] and for constipation at $2,306 [15], and similar to those for COPD [6].

Total ED and inpatient charges with hypertension diagnosis in any position (primary or secondary) were $406 billion in 2012. Concomitant hypertension in patients with diabetes is associated with higher healthcare utilization [16, 17]. Thus, the cost and morbidity burden of hypertension in the U.S. is quite significant. As a disease for which multiple treatment options are available, at least significant proportion of charges and burden may be preventable, by increasing patient awareness and knowledge, optimal medication adherence and access to outpatient clinics. These interventions may also further reduce the risk of associated complications, such as myocardial infarction, stroke etc. [3, 18].

We noted regional differences in health care utilization for hypertension regarding the risk of hospitalization, length of hospital stay, proportion discharged to home etc. Regional differences can be due to multiple reasons including due to regional differences in income, payment by insurers due to adjustment for cost-of-living and differences in health status [19]; health care intensity varies by region in the U.S. [20].

Our study identified several risk factors for higher ED charges with hypertension as the primary diagnosis. This fills a knowledge gap. Younger age, female sex, residence in metropolitan area, hospital location in South or West U.S. location, private insurance and the presence of HF were each associated with higher charges for an ED visit. Similarly, younger age, female sex, metropolitan residence, Medicaid insurance and hospital location in the Northeast, and co-existing diabetes, gout, CHD, COPD, hyperlipidemia and osteoarthritis were associated with a higher risk of hospitalization with hypertension as the primary diagnosis. Most comorbidities were associated with 1.5–3.3 fold higher risk of inpatient admission with hypertension as the primary diagnosis except renal failure and HF, which were associated with much higher odds of 7.5 and 8.6, respectively. Thus, we identified risk factors associated with higher rates of hospitalization (significantly more expensive than an ED visit alone) and higher charges. This paves the way for future studies that should examine whether optimization of associated comorbidities and/or recognition of high-risk patients with hypertension (based on socio-demographic characteristics) for frequent outpatient follow-up and implementation of special programs to improve access to outpatient health care teams can help reduce burden and costs associated with hypertension-associated ED and inpatient use. Our study further extends the recent finding that patients with hypertension with concomitant comorbidities are more likely to hospitalized compared to patients without comorbidities [12].

Another important observation from our study was that 11 % patients with inpatient utilization due to hypertension were discharged to skilled nursing/intermediate facilities and 13 % to home health care while 73 % were discharged to home. Older age, metropolitan residence and most comorbidities (except hyperlipidemia and gout) were associated with lower odds, whereas male sex, payer other than Medicare and South or West U.S. hospital location were associated with higher odds of discharge to home. The next step in this research should be to identify potential targets for intervention studies with an aim to reduce health care utilization and costs due to hypertension. It also remains to be seen whether optimization of key comorbidities we identified as risk factors can reduce morbidity and associated charges/costs.

We found that older age, metropolitan patient residence, metropolitan teaching hospital and Medicaid as primary payer and most comorbidities except hyperlipidemia and osteoarthritis were associated with longer stay whereas private insurance, hospital location other than Northeast were associated with shorter hospital stay. Similarly, older age, median household income in the highest quartile, metropolitan location and most comorbidities except hyperlipidemia and osteoarthritis were associated higher inpatient hospital charges. Uninsured primary payer and Midwest U.S. hospital location were associated with lower hospital charges.

Our findings must be interpreted considering study limitations. NEDS contains event-level data but not unique identifiers so that individuals may be represented by multiple visits in any given year. We were not able to examine readmission rates due to the lack of this information in NEDS. Study findings are likely not generalizable to other countries due to differences in health care settings. We suspect residual confounding due to the lack of availability of details regarding optimal vs. suboptimal control of hypertension [21] and the rate of adherence with anti-hypertensive medication [22], both of which are important factors for healthcare utilization. It is far more likely that these factors are causes/mediators of increased ED and inpatient utilization, rather than confounders. In addition, we are unaware of any data indicating that associations we noted are mediated through these factors. Therefore, even though the estimates may be possibly over- or under-estimates, the confounding bias is unclear/unknown. Outpatient utilization data were not available, and a study of hypertension associated complications such as stroke, myocardial infarction, renal failure etc. were beyond the scope of this study. Availability of only three years of data for our analyses did not allow us to have a very comprehensive look at time-trends; data from more years would have made this analysis more informative. However, some trends in cost very evident in this short time-period, as reported. Several regional differences were noted in various outcomes, but detailed analyses by region were outside the scope of this paper, and limited resources prevented us from doing these analyses.

Study strengths include the use of the largest ED and hospitalization data in the U.S. that is representative and generalizable to the U.S. population, inclusion of several important outcomes, and important covariates as potential predictors, and examination of data across several years.

## Conclusions

In summary, this contemporary study using national U.S. data provides a comprehensive assessment of ED and inpatient health care burden and charges due to hypertension in the U.S. The burden of hypertension is substantial and slowly increasing. We identified several important factors associated both with ED and inpatient charges for hypertension as well as disposition from ED and inpatient settings. Several of these factors may be modifiable and present potential targets for future interventions to reduce health care utilization and cost due to hypertension.

## Abbreviations

CHD, coronary heart disease; COPD, chronic obstructive pulmonary disease; ED, Emergency department; HF, heart failure; NEDS, National ED sample

## Additional files

Additional file 1: Patient Outcomes for ED visits with hypertension as the primary diagnosis. (DOC 37 kb) Additional file 2: Predictors of ED charges among patients presenting to ED with hypertension using linear regression. (DOC 59 kb) Additional file 3: Characteristics of patients with Hypertension ED visits with and without hospitalization. (DOC 56 kb) Additional file 4: Predictors of **Log of duration of hospital stay*** among patients with hypertension who were admitted to the hospital after presenting to ED with hypertension as the primary diagnosis using linear regression. (DOC 38 kb) Additional file 5: Predictors of log of duration of hospital stay among patients with hypertension who were admitted to the hospital after presenting to ED with hypertension as the primary diagnosis using linear regression. (DOC 59 kb) Additional file 6: Predictors of duration of hospital stay (>2 vs. ≤2 days) among patients with hypertension who were admitted to the hospital after presenting to ED with hypertension as the primary diagnosis using logistic regression. (DOC 62 kb) Additional file 7: Predictors of log of total hospital charges among patients who were admitted to the hospital after an ED visit with hypertension as the primary diagnosis using linear regression. (DOC 60 kb)
