# Supplementary material for: Emergency Department and Inpatient Healthcare utilization due to Hypertension
Source: BMC Health Serv Res. 2016 Jul 26;16:303. doi: 10.1186/s12913-016-1563-7 (PMC4962411; doi:10.1186/s12913-016-1563-7)
Supplement: Additional file 1: — Patient Outcomes for ED visits with hypertension as the primary diagnosis. (DOC 37 kb) [file 12913_2016_1563_MOESM1_ESM.doc]

**Supplementary file 1.** Patient Outcomes for ED visits with hypertension as the primary diagnosis

|  | **2009 NEDS** | **2010 NEDS** | **2012 NEDS** |
| --- | --- | --- | --- |
| ED disposition* |  |  |  |
| Discharged (routine) | 645,519 (70.09) | 692,355 (71.18) | 758,279 (72.83) |
| Admitted | 239,848 (26.04) | 243,071 (24.99) | 242,397 (23.28) |
| Transferred | 16,580 (1.80) | 20,003 (2.06) | 18,644 (1.98) |
| Home health care | 2,635 (0.29) | 1,098 (0.11) | 1,881 (0.18) |
| Against medical advice | 14,232 (1.55) | 13,386 (1.38) | 18,771 (1.80) |
| Unknown | 1,855 (0.20) | 2,264 (0.23) | 849 (0.08) |
| ED charges per ED visit, in US$, |  |  |  |
| Mean (SE) | 2,169.31 (54.07) | 2,333.58 (64.23) | 2,746.71 (75.92) |
| Median (IQR) | 1,382.93  (692.33, 2,554.98) | 1,499.92  (777.45, 2,726.76) | 1,758.37  (895.14, 3,215.50) |
| Length of hospital stay for admitted patients, in days |  |  |  |
| Mean (SE) | 4.13 (0.05) | 4.02 (0.05) | 3.98 (0.05) |
| Median (IQR) | 2.41 (1.16, 4.52) | 2.33 (1.12, 4.36) | 2.27 (1.07, 4.31) |
| Total charges per visit for admitted patients, US$) |  |  |  |
| Mean (SE) | 27,619 (763.31) | 29,177 (752.42) | 32,761 (894.63) |
| Median (IQR) | 17,633  (10,287, 31101) | 18,697  (11,080, 32887) | 20,820  (12,338, 36,699) |

IQR, inter-quartile range; SE, standard error
