# Supplementary material for: Emergency Department and Inpatient Healthcare utilization due to Hypertension
Source: BMC Health Serv Res. 2016 Jul 26;16:303. doi: 10.1186/s12913-016-1563-7 (PMC4962411; doi:10.1186/s12913-016-1563-7)
Supplement: Additional file 2: — Predictors of ED charges among patients presenting to ED with hypertension using linear regression. (DOC 59 kb) [file 12913_2016_1563_MOESM2_ESM.doc]

Supplementary file 2. Predictors of ED charges among patients presenting to ED with hypertension using linear regression

|  | Univariate |  | Multivariable-adjusted |  |
| --- | --- | --- | --- | --- |
|  | Beta-estimate* (95% CI) | P-value | Beta-estimate* (95% CI) | P-value |
| Age |  |  |  |  |
| <50 | Ref |  | Ref |  |
| 50- <65 | **112.90 (45.57,180.24)** | **0.0010** | 5.93 (-52.26, 64.13) | 0.8414 |
| 65- <80 | **214.52 (126.21, 302.83)** | **<0.0001** | -72.40 (-150.99, 6.18) | 0.0709 |
| ≥80 | **208.01 (95.61, 320.41)** | **0.0003** | **-102.71 (-197.98, -7.43)** | **0.0346** |
| Gender |  |  |  |  |
| Female (ref) | Ref |  | Ref |  |
| Male | **-196.61 (-239.55, -153.66)** | **<0.0001** | **-174.17 (-211.80, -136.53)** | **<0.0001** |
| Median household income |  |  |  |  |
| 1st quartile (< $38,999) | Ref |  | Ref |  |
| 2nd quartile ($39,000 to $47,999) | 107.99 (-23.58, 239.57) | 0.1075 | 26.75 (-103.37, 156.86) | 0.6867 |
| 3rd quartile ($48,000 to $62,999) | **386.86 (126.65, 647.07)** | **0.0036** | 206.26 (-21.61, 434.13) | 0.0760 |
| 4th quartile ($63,000 or more) | **332.79 (49.55, 616.03)** | **0.0214** | 162.16 (-129.92, 454.23) | 0.2761 |
| Primary payer |  |  |  |  |
| Medicare (ref) | Ref |  | Ref |  |
| Medicaid | **-273.85 (-408.72, -138.98)** | **0.0001** | **-184.39 (-292.77, -76.01)** | **0.0009** |
| Private insurance | 74.44 (-0.88, 149.76) | 0.0527 | 109.84 (17.53, 202.16) | 0.0198 |
| Self-pay/No charge | **-391.53 (-519.60, -263.47)** | **<0.0001** | **-304.33 (-434.29, -174.37)** | **<0.0001** |
| Other | **-459.26 (-821.98, -96.54)** | **0.0131** | **-383.85 (-686.43, -81.28)** | **0.0130** |
| Patient residence |  |  |  |  |
| Micropolitan/not metro | Ref |  | Ref |  |
| Metro (large or small) | **569.50 (366.16,7 72.85)** | **<0.0001** | **677.52 (453.39, 901.65)** | **<0.0001** |
| Hospital Region |  |  |  |  |
| Northeast | Ref |  | Ref |  |
| Midwest | 148.25 (-302.38, 598.88) | 0.5186 | 251.93 (-195.89, 699.75) | 0.2698 |
| South | 396.15 (-47.09, 839.40) | 0.0797 | **490.76 (13.61, 967.92)** | **0.0438** |
| West | **1321.51 (822.57, 1820.45)** | **<0.0001** | **1412.73 (903.57, 1921.89)** | **<0.0001** |
| Teaching status of hospital |  |  |  |  |
| Metropolitan non -teaching or non-metro | Ref |  | Ref |  |
| Metropolitan teaching | -101.71 (-404.84, 201.41) | 0.5103 | -181.94 (-522.44, 158.55) | 0.2945 |
| Comorbidities |  |  |  |  |
| CHD (ref: no) | **418.47 (299.34, 537.60)** | **<0.0001** | **251.66 (142.93, 360.39)** | **<0.0001** |
| Hyperlipidemia (ref: no) | **698.51 (570.00, 827.02)** | **<0.0001** | **581.39 (472.52, 690.26)** | **<0.0001** |
| Renal failure (ref: no) | **213.99 (85.30, 342.68)** | **0.0011** | **156.91 (35.20, 278.62)** | **0.0116** |
| Heart failure (ref: no) | -121.40 (-244.89, 2.09) | 0.0540 | **-511.12 (-618.03, -404.21)** | **<0.0001** |
| Gout (ref: no) | -162.23 (-353.90, 29.43) | 0.0970 | 10.19 (-163.40, 183.78) | 0.9083 |
| Diabetes (ref: no) | **303.56 (219.00, 388.12)** | **<0.0001** | **159.34 (91.21, 227.46)** | **<0.0001** |
| COPD (ref: no) | **332.08 (196.37, 467.78)** | **<0.0001** | **255.29 (133.16, 377.42)** | **<0.0001** |
| Osteoarthritis (ref: no) | **569.30 (365.35, 773.25)** | **<0.0001** | **347.33 (163.72, 530.94)** | **0.0002** |

CHD, coronary artery disease; COPD, chronic obstructive pulmonary disease.

**Significant beta coefficients are in bold**.
