# Supplementary material for: Emergency Department and Inpatient Healthcare utilization due to Hypertension
Source: BMC Health Serv Res. 2016 Jul 26;16:303. doi: 10.1186/s12913-016-1563-7 (PMC4962411; doi:10.1186/s12913-016-1563-7)
Supplement: Additional file 3: — Characteristics of patients with Hypertension ED visits with and without hospitalization. (DOC 56 kb) [file 12913_2016_1563_MOESM3_ESM.doc]

**Supplementary file 3**. Characteristics of patients with Hypertension ED visits with and without hospitalization

|  | **2012 NEDS (all)** | **2012 NEDS, Not admitted** | **2012 NEDS who were admitted** | **p-value, comparing not admitted vs. admitted** |
| --- | --- | --- | --- | --- |
| **ED visits, n (%)** | **1,041,223** | **798,826 (76.72%)** | **242,397 (23.28%)** |  |
| Age, in years |  |  |  | **<0.0001** |
| Mean (standard error) | **59.17 (0.22)** | **58.06 (0.22)** | **62.82 (0.28)** |  |
| Sex |  |  |  |  |
| Female | **595,212 (57.17)** | **464,086 (58.10)** | **131,125 (54.10)** |  |
| Patient location (residence) |  |  |  | **<0.0001** |
| Micropolitan/not metro | **184,852 (17.84)** | **156,689 (19.71)** | **28,164 (11.68)** |  |
| Metropolitan (large or small) | **851,485 (82.16)** | **638,447 (80.29)** | **213,038 (88.32)** |  |
| Median house hold income |  |  |  | **0.0067** |
| 1st quartile (< $38,999) | **389,354 (38.19)** | **299,375 (38.26)** | **89,979 (37.96)** |  |
| 2nd quartile ($39,000 to $47,999) | **255,324 (25.04)** | **198,849 (25.41)** | **56,475 (23.82)** |  |
| 3rd quartile ($48,000 to $62,999) | **219,568 (21.53)** | **168,286 (21.50)** | **51,281 (21.63)** |  |
| 4th quartile ($63,000 or more) | **155,369 (15.24)** | **116,042 (14.83)** | **39,327 (16.59)** |  |
| Primary payer |  |  |  | **<0.0001** |
| Medicare | **443,993 (42.69)** | **309,426 (38.79)** | **134,567 (55.55)** |  |
| Medicaid | **138,606 (13.33)** | **103,057 (12.92)** | **35,549 (14.67)** |  |
| Private insurance | **229,988 (22.12)** | **191,464 (24.00)** | **38,524 (15.90)** |  |
| Self-pay/No charge | **189,031 (18.18)** | **162,962 (20.43)** | **26,068 (10.76)** |  |
| Other | **38,337 (3.69)** | **30,797 (3.86)** | **7,540 (3.11)** |  |
| Hospital Region |  |  |  | **0.0006** |
| Northeast | **168,303 (16.16)** | **121,240 (15.18)** | **47,063 (19.42)** |  |
| Midwest | **219,784 (21.11)** | **170,550 (21.35)** | **49,234 (20.31)** |  |
| South | **490,154 (47.07)** | **380,195 (47.59)** | **109,959 (47.59)** |  |
| West | **162,982 (15.65)** | **126,841(15.88)** | **36,141 (14.91)** |  |
| Teaching status of hospital |  |  |  | **<0.0001** |
| Metropolitan non -teaching or non-metro | **576,146 (55.33)** | **456,604 (57.16)** | **119,542 (49.32)** |  |
| Metropolitan teaching | **465,077 (44.67)** | **342,221 (42.84)** | **122,856 (50.68)** |  |
| Comorbidities |  |  |  |  |
| CHD | **129,296 (12.42)** | **50,752 (6.35)** | **78,543 (32.40)** | **<0.0001** |
| Hyperlipidemia | **188,440 (18.10)** | **93,131 (11.66)** | **95,309 (39.32)** | **<0.0001** |
| Renal failure | **174,847 (16.79)** | **55,580 (6.96)** | **119,267 (49.20)** | **<0.0001** |
| Heart failure | **120,385 (11.56)** | **24,581 (3.08)** | **95,804 (39.52)** | **<0.0001** |
| Gout | **18,029 (1.73)** | **6,864 (0.86)** | **11,165 (4.61)** | **<0.0001** |
| Diabetes | **220,992 (21.22)** | **125,020 (15.65)** | **95,972 (39.59)** | **<0.0001** |
| COPD | **51,294 (4.93)** | **20,647 (2.58)** | **30,647 (12.64)** | **<0.0001** |
| Osteoarthritis | **24,894 (2.39)** | **9,539 (1.19)** | **15,354 (6.33)** | **<0.0001** |

CHD, coronary heart disease; COPD, chronic obstructive pulmonary disease;

*p-value comparing not admitted vs. admitted

**Statistically significant differences are in bold**
