# Supplementary material for: Emergency Department and Inpatient Healthcare utilization due to Hypertension
Source: BMC Health Serv Res. 2016 Jul 26;16:303. doi: 10.1186/s12913-016-1563-7 (PMC4962411; doi:10.1186/s12913-016-1563-7)
Supplement: Additional file 4: — Predictors of Log of duration of hospital stay* among patients with hypertension who were admitted to the hospital after presenting to ED with hypertension as the primary diagnosis using linear regression. (DOC 38 kb) [file 12913_2016_1563_MOESM4_ESM.doc]

**Supplementary file 4.** Discharge disposition of patients after hospital admission with hypertension as the primary diagnosis, after an ED visit for hypertension

|  | **2009 NEDS** | **2010 NEDS** | **2012 NEDS** |
| --- | --- | --- | --- |
| **Duration of hospital stay, in days,** Mean (SE) |  |  |  |
| Hypertension as primary for those with ED visits | 4.13 (0.05) | 4.02 (0.05) | 3.98 (0.05) |
| Hypertension primary or secondary diagnosis for ED visit | 4.93 (0.04) | 4.81 (0.03) | 4.72 (0.04) |
| **Hospitalization disposition for hypertension as primary diagnosis,** n (%) |  |  |  |
| Discharged home | 176,601 (73.65) | 177,574 (73.15) | 173,571 (71.61) |
| Skilled nursing facility, intermediate Care facility, and another type of facility | 25,682 (10.71) | 24,708 (10.18) | 25,944 (10.70) |
| Transferred to short term hospital | 4,083 (1.70) | 4,021 (1.66) | 3,651 (1.51) |
| Home health care | 25,288 (10.55) | 28,109 (11.58) | 30,582 (12.62) |
| Against medical advice | 5,535 (2.31) | 5,635 (2.32) | 6,059 (2.50) |
| Died | 2,496 (1.04) | 2,555 (1.05) | 2,512 (1.04) |
| **Hospitalization disposition with hypertension as primary or secondary diagnosis*,** n (%) |  |  |  |
| Discharged home | 5,989,448 (58.40) | 6,069,191 (57.88) | 5,952,200 (57.62) |
| Skilled nursing facility, intermediate Care facility, and another type of facility | 2,240,739 (21.85) | 2,282,869 (21.77) | 2,239,045 (21.67) |
| Transferred to short term hospital | 304,343 (2.97) | 298,744 (2.85) | 273,834 (2.65) |
| Home health care | 1,311,794 (12.79) | 1,429,979 (13.64) | 1,476,112 (14.29) |
| Against medical advice | 127,416 (1.24) | 131,366 (1.25) | 140,662 (1.36) |
| Died | 274,809 (2.68) | 266,296 (2.54) | 244,252 (2.36) |

SE, standard error
