# Supplementary material for: Emergency Department and Inpatient Healthcare utilization due to Hypertension
Source: BMC Health Serv Res. 2016 Jul 26;16:303. doi: 10.1186/s12913-016-1563-7 (PMC4962411; doi:10.1186/s12913-016-1563-7)
Supplement: Additional file 5: — Predictors of log of duration of hospital stay among patients with hypertension who were admitted to the hospital after presenting to ED with hypertension as the primary diagnosis using linear regression. (DOC 59 kb) [file 12913_2016_1563_MOESM5_ESM.doc]

**Supplementary file 5**. Predictors of log of duration of hospital stay* among patients with hypertension who were admitted to the hospital after presenting to ED with hypertension as the primary diagnosis using linear regression

|  | Univariate |  | Multivariable-adjusted |  |
| --- | --- | --- | --- | --- |
|  | B-estimate (95% CI) | P-value | B-estimate (95% CI) | P-value |
| Age |  |  |  |  |
| <50 | Ref |  | Ref |  |
| 50- <65 | **0.11 (0.08, 0.13)** | **<0.0001** | **0.08 (0.06, 0.10)** | **<0.0001** |
| 65- <80 | **0.20 (0.17, 0.22)** | **<0.0001** | **0.12 (0.10, 0.14)** | **<0.0001** |
| ≥80 | **0.26 (0.23, 0.29)** | **<0.0001** | **0.17 (0.14, 0.19)** | **<0.0001** |
| Gender |  |  |  |  |
| Female (ref) | Ref |  | Ref |  |
| Male | 0.01 (-0.01, 0.02) | 0.4440 | **-0.02 (-0.04, -0.01)** | **0.0003** |
| Median house hold income |  |  |  |  |
| 1st quartile (< $38,999) | Ref |  | Ref |  |
| 2nd quartile ($39,000 to $47,999) | 0.01 (-0.01, 0.03) | 0.4520 | 0.01 (-0.01, 0.03) | 0.2699 |
| 3rd quartile ($48,000 to $62,999) | 0.01 (-0.02, 0.04) | 0.7043 | 0.00 (-0.03, 0.03) | 0.9516 |
| 4th quartile ($63,000 or more) | 0.04 (0.00, 0.07) | 0.0357 | 0.00 (-0.03, 0.03) | 0.9040 |
| Primary payer |  |  |  |  |
| Medicare (ref) | Ref |  | Ref |  |
| Medicaid | **-0.08 (-0.11, -0.05)** | **<0.0001** | **0.04 (0.01, 0.06)** | **0.0184** |
| Private insurance | **-0.20 (-0.22, -0.18)** | **<0.0001** | **-0.02 (-0.04, -0.01)** | **0.0094** |
| Self-pay/No charge | **-0.27 (-0.30, -0.25)** | **<0.0001** | **-0.05 (-0.08, -0.03)** | **0.0001** |
| Other | **-0.18 (-0.28, -0.09)** | **0.0001** | -0.00 (-0.10, 0.09) | 0.9365 |
| Patient location (residence) |  |  |  |  |
| Micropolitan/not metro | Ref |  | Ref |  |
| Metro (large or small) | **0.04 (0.00, 0.07)** | **0.0402** | 0.02 (-0.00, 0.05) | 0.0866 |
| Hospital Region |  |  |  |  |
| Northeast | Ref |  | Ref |  |
| Midwest | **-0.12 (-0.18, -0.05)** | **0.0004** | **-0.13 (-0.19, -0.08)** | **<0.0001** |
| South | **-0.07 (-0.12, -0.02)** | **0.0038** | **-0.08 (-0.12, -0.04)** | **0.0001** |
| West | **-0.08 (-0.13, -0.02)** | **0.0060** | **-0.13 (-0.18, -0.08)** | **<0.0001** |
| Teaching status of hospital |  |  |  |  |
| Metropolitan non -teaching or non-metro | Ref |  | Ref |  |
| Metropolitan teaching | 0.01 (-0.02, 0.05) | 0.5138 | 0.01 (-0.02, 0.04) | 0.4486 |
| Comorbidities |  |  |  |  |
| CHD (ref: no) | **0.14 (0.12, 0.15)** | **<0.0001** | **0.01 (0.00, 0.03)** | **0.0362** |
| Hyperlipidemia (ref: no) | **-0.04 (-0.06, -0.03)** | **<0.0001** | **-0.07 (-0.08, -0.06)** | **<0.0001** |
| Renal failure (ref: no) | **0.36 (0.34, 0.39)** | **<0.0001** | **0.28 (0.25, 0.30)** | **<0.0001** |
| Heart failure (ref: no) | **0.39 (0.37, 0.41)** | **<0.0001** | **0.28 (0.26, 0.30)** | **<0.0001** |
| Gout (ref: no) | **-0.11 (-0.14, -0.08)** | **<0.0001** | 0.00 (-0.02, 0.03) | 0.7419 |
| Diabetes (ref: no) | **0.14 (0.12, 0.15)** | **<0.0001** | **0.05 (0.04, 0.06)** | **<0.0001** |
| COPD (ref: no) | **0.21 (0.18, 0.23)** | **<0.0001** | **0.06 (0.05, 0.08)** | **<0.0001** |
| Osteoarthritis (ref: no) | 0.02 (-0.00, 0.05) | 0.0717 | 0.00 (-0.02, 0.03) | 0.7757 |

CHD, coronary heart disease; COPD, chronic obstructive pulmonary disease.

**Significant beta coefficients are in bold**.

*Log of duration of hospital stay was examined since this was distributed more normally than the hospital stay variable
