# Supplementary material for: Emergency Department and Inpatient Healthcare utilization due to Hypertension
Source: BMC Health Serv Res. 2016 Jul 26;16:303. doi: 10.1186/s12913-016-1563-7 (PMC4962411; doi:10.1186/s12913-016-1563-7)
Supplement: Additional file 6: — Predictors of duration of hospital stay (>2 vs. ≤2 days) among patients with hypertension who were admitted to the hospital after presenting to ED with hypertension as the primary diagnosis using logistic regression. (DOC 62 kb) [file 12913_2016_1563_MOESM6_ESM.doc]

**Supplementary file 6.** Predictors of duration of hospital stay >2 days (reference, ≤2 days) among patients with hypertension who were admitted to the hospital after presenting to ED with hypertension as the primary diagnosis using logistic regression

|  | Univariate |  | Multivariable-adjusted |  |
| --- | --- | --- | --- | --- |
|  | B-estimate (95% CI) | P-value | B-estimate (95% CI) | P-value |
| Age |  |  |  |  |
| <50 | Ref |  | Ref |  |
| 50- <65 | 1.33 (1.26, 1.40) | **<0.0001** | 1.22 (1.15, 1.30) | **<0.0001** |
| 65- <80 | 1.71 (1.60, 1.82) | **<0.0001** | 1.34 (1.24, 1.44) | **<0.0001** |
| ≥80 | 2.24 (2.08, 2.41) | **<0.0001** | 1.73 (1.60, 1.88) | **<0.0001** |
| Gender |  |  |  |  |
| Female | Ref |  | Ref |  |
| Male | 1.01 (0.97, 1.05) | 0.6252 | 0.93 (0.89, 0.97) | **0.0009** |
| Median house hold income |  |  |  |  |
| 1st quartile (< $38,999) | Ref |  | Ref |  |
| 2nd quartile ($39,000 to $47,999) | 1.03 (0.96, 1.11) | 0.4396 | 1.04 (0.96, 1.11) | 0.3318 |
| 3rd quartile ($48,000 to $62,999) | 1.00 (0.91, 1.09) | 0.9427 | 0.98 (0.89, 1.07) | 0.5844 |
| 4th quartile ($63,000 or more) | 1.07 (0.97, 1.18) | 0.1914 | 0.97 (0.88, 1.07) | 0.5851 |
| Primary payer |  |  |  |  |
| Medicare | Ref |  | Ref |  |
| Medicaid | 0.75 (0.70, 0.80) | **<0.0001** | 1.04 (0.96, 1.14) | 0.3259 |
| Private insurance | 0.54 (0.51, 0.58) | **<0.0001** | **0.89 (0.84, 0.96)** | **0.0011** |
| Self-pay/No charge | 0.43 (0.40, 0.47) | **<0.0001** | 0.80 (0.73, 0.88) | **<0.0001** |
| Other | 0.62 (0.51, 0.75) | **<0.0001** | 1.05 (0.85, 1.30) | 0.6459 |
| Patient location (residence) |  |  |  |  |
| Micropolitan/ not metro | Ref |  | Ref |  |
| Metro (large or small) | 1.09 (0.98, 1.21) | 0.1022 | 1.09 (1.00, 1.20) | 0.0568 |
| Hospital Region |  |  |  |  |
| Northeast | Ref |  | Ref |  |
| Midwest | **0.81 (0.68, 0.97)** | **0.0221** | **0.74 (0.62, 0.87)** | **0.0004** |
| South | **0.87 (0.76, 0.99)** | **0.0406** | **0.81 (0.72, 0.91)** | **0.0007** |
| West | **0.86 (0.74, 1.00)** | **0.0495** | **0.69 (0.60, 0.80)** | **<0.0001** |
| Teaching status of hospital |  |  |  |  |
| Metropolitan non -teaching or non-metro | Ref |  | Ref |  |
| Metropolitan teaching | 1.00 (0.91, 1.10) | 0.9874 | 1.00 (0.92, 1.10) | 0.9573 |
| Comorbidities |  |  |  |  |
| CHD (ref: no) | 1.55 (1.48, 1.63) | **<0.0001** | **1.08 (1.03, 1.13)** | **0.0017** |
| Hyperlipidemia (ref: no) | 0.96 (0.92, 1.00) | 0.0503 | 0.85 (0.82, 0.89) | **<0.0001** |
| Renal failure (ref: no) | 2.83 (2.66, 3.01) | **<0.0001** | 2.29 (2.16, 2.43) | **<0.0001** |
| Heart failure (ref: no) | 3.17 (2.99, 3.35) | **<0.0001** | 2.35 (2.22, 2.48) | **<0.0001** |
| Gout (ref: no) | 1.45 (1.33, 1.57) | **<0.0001** | 1.03 (0.95, 1.13) | 0.4575 |
| Diabetes (ref: no) | 1.57 (1.50, 1.63) | **<0.0001** | 1.26 (1.20, 1.31) | **<0.0001** |
| COPD (ref: no) | 1.84 (1.73, 1.96) | **<0.0001** | 1.24 (1.17, 1.32) | **<0.0001** |
| OA (ref: no) | 1.18 (1.09, 1.28) | **<0.0001** | **1.10 (1.01, 1.19)** | **0.0251** |

CHD, coronary heart disease; COPD, chronic obstructive pulmonary disease;

**Significant odds ratios are in bold**.
